# Sevoflurane versus propofol and the long‐term risk of attention‐deficit/hyperactivity disorder in children

**DOI:** 10.1002/gps3.70000

**Published:** 2026-04-16

**Authors:** Mingyang Sun, Yangyang Wang, Yuqin Tang, Peilin Xie, Tian Mao, Zhongyuan Lu, Jiao Wang, Liang Zhao, Saihao Fu, Mengrong Miao, Wan‐Ming Chen, Szu‐Yuan Wu, Jiaqiang Zhang

**Affiliations:** ^1^ Department of Anesthesiology and Perioperative Medicine People's Hospital of Zhengzhou University Henan Provincial People's Hospital Zhengzhou Henan China; ^2^ Institute of Electrophysiology Henan Academy of Innovations in Medical Science Zhengzhou Henan China; ^3^ Academy of Medical Sciences of Zhengzhou University Zhengzhou Henan China; ^4^ Graduate Institute of Business Administration College of Management Fu Jen Catholic University Taipei Taiwan China; ^5^ Artificial Intelligence Development Center Fu Jen Catholic University Taipei Taiwan China; ^6^ Department of Food Nutrition and Health Biotechnology, College of Medical and Health Science Asia University Taichung Taiwan China; ^7^ Big Data Center Lo‐Hsu Medical Foundation Lotung Poh‐Ai Hospital Yilan Taiwan China; ^8^ Division of Radiation Oncology Lo‐Hsu Medical Foundation Lotung Poh‐Ai Hospital Yilan Taiwan China; ^9^ Department of Healthcare Administration, College of Medical and Health Science Asia University Taichung Taiwan China; ^10^ Cancer Center Lo‐Hsu Medical Foundation Lotung Poh‐Ai Hospital Yilan Taiwan China; ^11^ Centers for Regional Anesthesia and Pain Medicine Taipei Municipal Wan Fang Hospital Taipei Medical University Taipei Taiwan China

**Keywords:** attention‐deficit/hyperactivity disorder (ADHD), neurodevelopment, paediatric anaesthesia, propofol, sevoflurane

## Abstract

**Background:**

Preclinical studies have shown that volatile anaesthetics, particularly sevoflurane, can disrupt neurodevelopment by inducing neuronal apoptosis, neuroinflammation and altered synaptic plasticity during critical periods of brain maturation. Whether these mechanisms translate into long‐term neurobehavioral risk in children remains uncertain.

**Aims:**

To compare the long‐term risk of attention‐deficit/hyperactivity disorder (ADHD) following paediatric anaesthesia with sevoflurane versus propofol in a large, multinational real‐world cohort.

**Methods:**

We conducted a large, multinational, retrospective cohort study using real‐world electronic health record data from more than 150 healthcare organisations across North America, Europe and Asia. Children and adolescents (0–18 years) who underwent a single surgical procedure under general anaesthesia between 2005 and 2025 were included. Patients with ADHD or multiple anaesthetic exposures were excluded. The primary exposure was sevoflurane versus propofol as the main anaesthetic. The primary outcome was new‐onset ADHD identified by International Classification of Diseases, Ninth or Tenth Revision codes after surgery. Propensity‐score matching (1:1), subgroup, sensitivity and positive/negative control analyses were performed to ensure robustness.

**Results:**

Among 54 102 matched children (27 051 per group), the cumulative incidence of ADHD was 5.63% after sevoflurane and 2.95% after propofol, corresponding to incidence rates of 134.9 and 105.4 per 10 000 person‐years. Sevoflurane exposure was associated with a higher risk of ADHD (hazard ratio 1.21; 95% confidence interval 1.11–1.31; *p* < 0.001). Findings were consistent across subgroups and sensitivity analyses; mortality was rare and similar between groups.

**Conclusions:**

In this multinational cohort, sevoflurane exposure during paediatric anaesthesia was associated with an increased long‐term risk of ADHD compared with propofol. These findings suggest that anaesthetic choice may have enduring neurobehavioral consequences and that prospective validation is warranted to guide safer paediatric anaesthesia practice.

## Abstract (highlights)

**WHAT IS ALREADY KNOWN ON THIS TOPIC**

- Preclinical and clinical studies have raised concerns that exposure to general anaesthetics during childhood may affect long‐term neurodevelopment. However, evidence directly comparing specific anaesthetic agents remains limited.

**WHAT THIS STUDY ADDS**

- In this multinational propensity score‐matched cohort of children undergoing a single surgical procedure, sevoflurane exposure was associated with a higher long‐term risk of attention‐deficit/hyperactivity disorder (ADHD) than propofol exposure. The observed association remained directionally consistent across subgroup and sensitivity analyses.

**HOW THIS STUDY MIGHT AFFECT RESEARCH, PRACTICE OR POLICY**

- These findings support greater attention to agent‐specific neurodevelopmental safety when selecting anaesthetic strategies for children and provide a rationale for future prospective studies to inform clinical practice and policy.

**Abbreviations**
ADHD: attention‐deficit/hyperactivity disorder
aHR: adjusted hazard ratio
ASMD: absolute standardised mean difference
BMI: body mass index
CI: confidence interval
EHR: electronic health record
E‐value: evidence value
GABA_A: gamma‐aminobutyric acid type A receptor
GAS: General Anaesthesia compared to Spinal anaesthesia Trial
HR: hazard ratio
ICD: International Classification of Diseases
IQR: interquartile range
LoRD‐KNeA: low‐dose radiation for knee osteoarthritis trial
MASK: Mayo Anaesthesia Safety in Kids Study
NMDA: *N*‐methyl‐D‐aspartate receptor
PANDA: Pediatric Anaesthesia NeuroDevelopment Assessment Study
PSM: propensity score matching
RCT: randomised controlled trial
SD: standard deviation
T4: thyroxine
TriNetX: TriNetX Global Collaborative Network
TSH: thyroid‐stimulating hormone
VAS: visual analogue scale

## INTRODUCTION

Each year, millions of children worldwide undergo surgical or diagnostic procedures requiring general anaesthesia, making anaesthetic exposure an almost unavoidable component of modern paediatric care.^1^, ^2^, ^3^ Over the past two decades, converging animal and translational evidence has raised growing concern that commonly used anaesthetic agents may exert lasting deleterious effects on the developing brain.^4^, ^5^ Preclinical studies have demonstrated that volatile inhalational anaesthetics—particularly sevoflurane—can induce neuronal apoptosis, impair synaptic plasticity and trigger neuroinflammatory cascades during critical periods of synaptogenesis.^6^, ^7^, ^8^, ^9^ These neurobiologic perturbations have been linked to persistent cognitive and behavioural impairments, including hyperactivity and inattention phenotypes analogous to attention‐deficit/hyperactivity disorder (ADHD).^10^, ^11^ Recent neuropsychiatric research further conceptualises ADHD as a disorder of aberrant neural network maturation and attentional control, characterised by altered cortical information processing, impaired deployment of attention and disrupted event segmentation mechanisms.^12^

Despite these mechanistic insights, evidence in humans remains inconsistent and fragmentary. Most clinical studies have been small, limited by short follow‐up, heterogeneous designs and an inability to disentangle the neurotoxic effects of anaesthetic agents from those of surgery, perioperative stress or preexisting vulnerabilities.^13^, ^14^, ^15^, ^16^, ^17^ Furthermore, few investigations have compared the neurodevelopmental safety of specific anaesthetics, leaving clinicians uncertain about which agents pose greater risk to the developing brain.^13^, ^14^, ^15^, ^16^, ^17^, ^18^, ^19^ This knowledge gap has sustained a long‐standing clinical question: Does exposure to different anaesthetic agents confer differential risks for long‐term neurobehavioral disorders in children?

Among modern anaesthetics, sevoflurane remains the most widely used volatile agent for paediatric induction and maintenance, whereas propofol, a short‐acting intravenous γ‐aminobutyric acid type A (GABA_A_) receptor modulator, serves as the principal alternative.^20^, ^21^ Although both achieve comparable clinical depth of anaesthesia, their mechanisms differ profoundly. Sevoflurane acts as a multi‐ion channel inhibitor, simultaneously antagonising *N*‐methyl‐D‐aspartate (NMDA) receptors and potentiating GABA_A_ receptors—a dual mechanism implicated in widespread apoptotic neurodegeneration and disruption of cortical circuit maturation.^22^, ^23^ In contrast, propofol's selective GABA_A_ modulation, rapid metabolism and limited NMDA interference are thought to attenuate excitotoxic and neuroinflammatory processes, suggesting a potentially safer neurodevelopmental profile.^24^, ^25^

To address this critical gap, we conducted a large‐scale, multinational retrospective cohort study using real‐world data to compare long‐term neurobehavioural outcomes between children exposed to sevoflurane and those exposed to propofol. By integrating propensity‐score matching (PSM), sensitivity analyses and outcome‐specific validation using positive and negative controls, this study provides the first real‐world, head‐to‐head comparison of these two anaesthetic agents in paediatric surgery. Our findings aim to clarify their differential impact on subsequent ADHD risk and inform evidence‐based anaesthetic selection to enhance neurodevelopmental safety in children.

## METHODS

### Study population and data source

We conducted a retrospective cohort study using the TriNetX Global Collaborative Network, a federated electronic health record platform encompassing more than 150 healthcare organisations across North America, Europe and Asia.^26^, ^27^, ^28^, ^29^ Children and adolescents aged 0–18 years who underwent surgical procedures requiring general anaesthesia between September 2005 and September 2025 were eligible. The index date was defined as the date of the first qualifying anaesthetic exposure during the study period. To ensure adequate covariate assessment, patients were required to have at least 12 months of continuous medical records before the index date. The cohort assembly process, including data source identification, inclusion and exclusion criteria, exposure classification, PSM and derivation of the final analytic cohorts, is summarised in figure S1. This study was conducted using de‐identified and aggregated data obtained from the TriNetX Global Collaborative Network. In accordance with the US Health Insurance Portability and Accountability Act, analyses of such data are considered exempt from the institutional review board review under 45 Code of Federal Regulations §164.514.

To enhance exposure classification and minimise residual confounding, we applied several exclusion criteria. Patients were excluded if they had a prior diagnosis of ADHD, received both sevoflurane and propofol on the index date or underwent more than one qualifying surgery during the study period. We also excluded individuals with incomplete demographic or baseline information or with < 6 months of follow‐up after the index date to ensure valid outcome ascertainment.

### Exposure definition

The exposure of interest was the primary anaesthetic administered during the index surgery. Patients were classified into two mutually exclusive groups: those who received inhaled sevoflurane or those who received intravenous propofol. Exposure status was determined solely by the anaesthetic administered at the first qualifying surgery.

To isolate the neurodevelopmental effects attributable to a single anaesthetic exposure and to avoid bias related to cumulative dose, surgical complexity, or repeated anaesthetic exposures, we restricted the cohort to children undergoing one qualifying operation during the study period. Patients with multiple anaesthesia records or more than one eligible surgery were excluded a priori. This restriction ensured that both exposure groups represented children with a single, well‐defined anaesthetic exposure, minimising confounding from repeated procedures that might independently increase the risk of postoperative neurobehavioural changes such as ADHD.

### Outcomes

The primary outcome was new‐onset ADHD, identified using International Classification of Diseases, Ninth or Tenth Revision (ICD‐9: 314; ICD‐10: F90) diagnostic codes recorded after the index date. To improve specificity, supplemental definitions were applied in sensitivity analyses, requiring either (1) at least two ADHD‐coded encounters, (2) a diagnosis combined with a prescription for stimulant medications or (3) a diagnosis established in a child psychiatry encounter.

**FIGURE 1:**
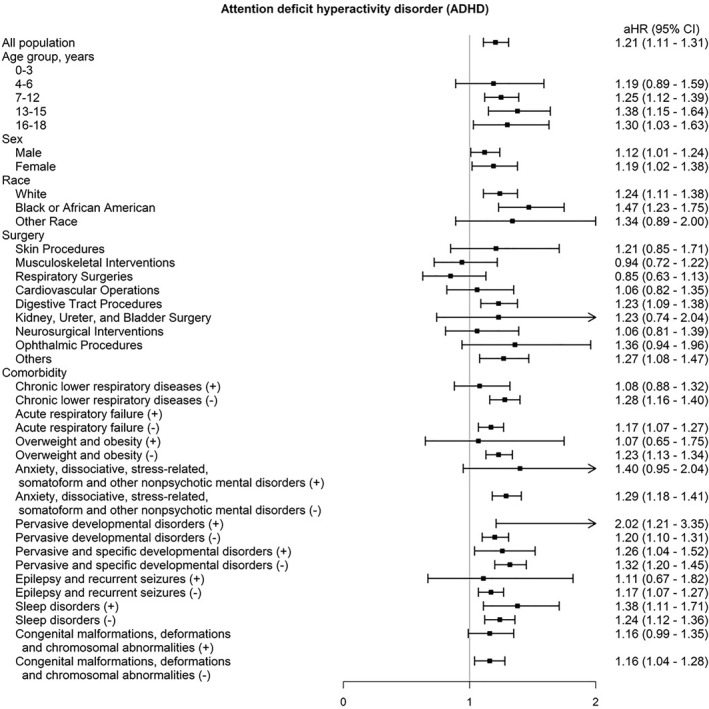
Subgroup analyses of the association between sevoflurane exposure and risk of attention‐deficit/hyperactivity disorder in children. aHR, adjusted hazard ratio; CI, confidence interval.

Secondary outcomes included all‐cause mortality and neuropsychiatric disorders plausibly associated with anaesthetic exposure, specifically anxiety and depression. Positive and negative control outcomes were prespecified to evaluate residual bias: delirium was analysed as a positive control, whereas common cold and irritable bowel syndrome served as negative controls. Subgroup analyses stratified by demographic factors, surgical categories and comorbidities are presented in figure 1, with consistent associations observed across most strata.

### Covariates

Baseline covariates were selected a priori based on three prespecified principles: (1) biological and clinical plausibility for influencing neurodevelopmental outcomes, (2) prior literature on paediatric anaesthesia and ADHD risk and (3) availability and completeness within the electronic health record system. Baseline covariates were assessed during the 12 months preceding the index date and included demographic characteristics (age, sex, race and ethnicity), comorbidities (such as chronic lower respiratory diseases, epilepsy, sleep disorders, congenital malformations, perinatal conditions and developmental or psychiatric disorders) and laboratory or nutritional biomarkers (body mass index [BMI], thyroid function [thyroid‐stimulating hormone and free T4], 25‐hydroxyvitamin D, ferritin, blood lead, zinc and magnesium). All selected covariates were measured before anaesthesia exposure and were entered simultaneously into the propensity‐score model. Balance between treatment groups was evaluated using absolute standardised mean differences (ASMD), with values < 0.1 considered acceptable.

### Subgroup analyses

We performed prespecified subgroup analyses to evaluate the consistency of the association across clinically relevant strata. Subgroups were defined by age category (0–3, 4–6, 7–12, 13–15 and 16–18 years), sex and race or ethnicity (White, Black or African American and other). Additional stratification was conducted by surgical type (skin, musculoskeletal, respiratory, cardiovascular, digestive tract, neurosurgical, ophthalmic and other procedures) and by key comorbid conditions (chronic respiratory disease, epilepsy, sleep disorders and developmental or congenital abnormalities). Exploratory analyses further stratified patients according to abnormal laboratory biomarkers (e.g., thyroid dysfunction, vitamin D deficiency, ferritin depletion or abnormal trace metals). Hazard ratios (HRs) were estimated within each subgroup, and interaction tests were conducted to assess heterogeneity.

### Sensitivity analyses

We conducted multiple sensitivity analyses to test the robustness of our findings. To address short‐term ascertainment bias, follow‐up initiation was deferred to 18 and 24 months after the index surgery; the elevated risk of ADHD associated with sevoflurane remained (table S1). To mitigate bias from incomplete observation, analyses were restricted to patients with at least 2 or 3 years of continuous follow‐up, yielding consistent HRs (table S2). Robustness to propensity score specifications was assessed by applying alternative calliper widths and overlap trimming, with results that were stable across different matching parameters (table S3). We further tested the definition of ADHD by requiring at least two diagnostic codes, ADHD plus stimulant prescription or ADHD diagnosis in a child psychiatry encounter; across these case definitions, the association persisted with comparable effect estimates (table S4). To reduce confounding from baseline neurodevelopmental vulnerabilities, we repeated the analyses after excluding children with congenital/chromosomal disorders, epilepsy/seizures or perinatal complications; HRs were again consistent, supporting the robustness of the observed association (table S5). Finally, quantitative bias analysis using E‐values suggested that only a confounder with a strong association with both exposure and outcome could fully explain the observed association (table S6).

### Statistical analysis

**FIGURE 2:**
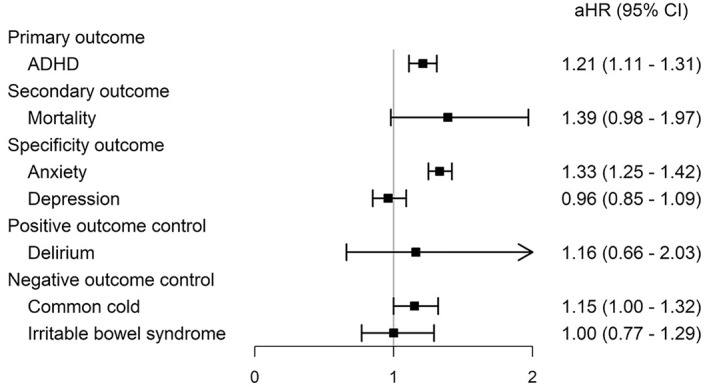
Validation analyses of sevoflurane exposure and ADHD risk using secondary, specificity and control outcomes. ADHD, attention‐deficit/hyperactivity disorder. aHR, adjusted hazard ratio; CI, confidence interval.

**FIGURE 3:**
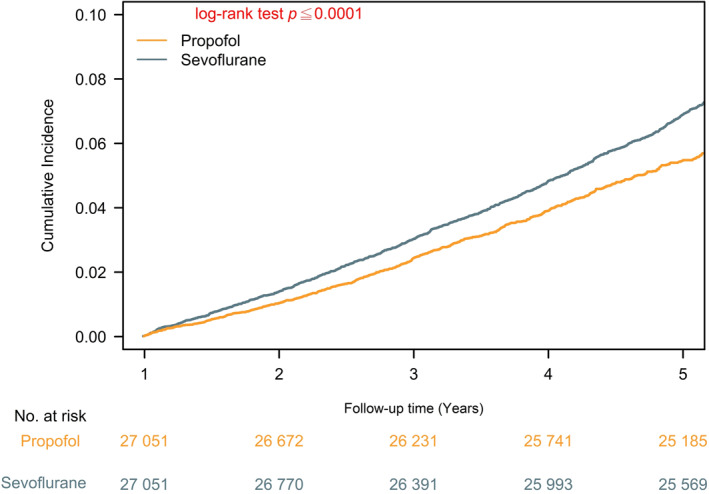
Cumulative incidence of attention‐deficit/hyperactivity disorder in children exposed to sevoflurane versus propofol.

PSM was conducted in a 1 : 1 ratio using nearest‐neighbour matching with a calliper width of 0.2 of the standard deviation of the logit of the propensity score. After matching, covariate balance was confirmed with ASMD < 0.1 across all variables (table 1). To further address residual confounding beyond matching, multivariable Cox proportional hazards models were fitted within the matched cohorts, adjusting for all baseline covariates included in the propensity‐score model. HRs and 95% confidence intervals (CIs) were estimated using Cox proportional hazards models. Kaplan–Meier survival curves were generated to compare ADHD incidence and all‐cause mortality, with log‐rank tests applied for between‐group differences (figures 1, 2, 3). Incidence rates per 10 000 person‐years, risk differences, risk ratios and odds ratios with 95% CIs were also reported (table 2). Residual bias and unmeasured confounding were further evaluated through prespecified sensitivity analyses, positive and negative control outcomes and quantitative bias analysis using E‐values. All statistical tests were two‐sided, with *p* values < 0.05 considered statistically significant.

**TABLE 1:** Baseline characteristics by anaesthetic exposure in paediatric surgery (sevoflurane vs. propofol), with standardised mean differences before and after propensity‐score matching

Covariate	Before PSM					After PSM				
	Sevoflurane		Propofol		ASMD	Sevoflurane		Propofol		ASMD
Age at index, mean (SD)	4.92	(4.33)	6.36	(4.73)	0.3169	4.92	(4.33)	4.95	(4.34)	0.0053
Sex, *n* (%)										
Male	15 445	(57.10)	554 313	(56.77)	0.0066	15 445	(57.1)	15 450	(57.11)	0.0004
Female	11 595	(42.86)	416 720	(42.68)	0.0038	11 595	(42.86)	11 585	(42.83)	0.0007
Unknown gender	11	(0.04)	5443	(0.56)	0.0947	11	(0.04)	16	(0.06)	0.0083
Race, *n* (%)										
White	15 952	(58.97)	594 510	(60.88)	0.0390	15 952	(58.97)	15 993	(59.12)	0.0031
Black or African American	6180	(22.85)	142 968	(14.64)	0.2114	6180	(22.85)	6197	(22.91)	0.0015
Asian	476	(1.76)	39 540	(4.05)	0.1367	476	(1.76)	461	(1.70)	0.0043
Other race	1881	(6.95)	91 870	(9.41)	0.0897	1881	(6.95)	1902	(7.03)	0.0030
Unknown race	2415	(8.93)	95 226	(9.75)	0.0283	2415	(8.93)	2354	(8.70)	0.0080
Coexisting comorbidities, *n* (%)										
Chronic lower respiratory diseases	2672	(9.88)	68 234	(6.99)	0.1041	2672	(9.88)	2684	(9.92)	0.0015
Acute respiratory failure	555	(2.05)	15 482	(1.59)	0.0349	555	(2.05)	492	(1.82)	0.0169
Cerebrovascular diseases	238	(0.88)	7145	(0.73)	0.0166	238	(0.88)	185.0	(0.68)	0.0222
Hydrocephalus	280	(1.04)	7557	(0.77)	0.0276	280	(1.04)	256	(0.95)	0.0090
Perinatal conditions	2198	(8.13)	51 873	(5.31)	0.1125	2198	(8.13)	2191	(8.10)	0.0009
Congenital malformations/chromosomal	6935	(25.64)	200 105	(20.49)	0.1223	6935	(25.64)	6818	(25.20)	0.0099
Mood disorders	154	(0.57)	7711	(0.79)	0.0268	154	(0.57)	161	(0.60)	0.0034
Anxiety & related disorders	998	(3.69)	35 096	(3.59)	0.0051	998	(3.69)	975	(3.60)	0.0045
Obsessive‐compulsive disorder	28	(0.10)	905	(0.09)	0.0035	28	(0.10)	28	(0.10)	0.0000
Intellectual disabilities	101	(0.37)	3422	(0.35)	0.0038	101	(0.37)	100	(0.37)	0.0006
Pervasive developmental disorders	393	(1.45)	17 379	(1.78)	0.0259	393	(1.45)	384	(1.42)	0.0028
Specific developmental disorders	2451	(9.06)	77 147	(7.90)	0.0417	2451	(9.06)	2444	(9.04)	0.0009
Epilepsy/recurrent seizures	554	(2.05)	17 804	(1.82)	0.0163	554	(2.05)	521	(1.93)	0.0087
Sleep disorders	2922	(10.80)	126 710	(12.98)	0.0672	2922	(10.80)	2925	(10.81)	0.0004
Laboratory and nutritional biomarkers, mean (SD)										
BMI (kg/m^2^)	18.33	(5.46)	19.51	(5.76)	0.2109	18.33	(5.46)	18.40	(5.21)	0.0130
TSH (mIU/L)	2.62	(3.23)	2.96	(19.17)	0.0251	2.62	(3.23)	2.57	(1.96)	0.0189
Free T4 (ng/dL)	1.18	(0.31)	1.18	(0.31)	0.0078	1.18	(0.31)	1.18	(0.31)	0.0162
25‐OH vitamin D (ng/mL)	30.80	(15.82)	32.21	(16.14)	0.0880	30.80	(15.82)	30.53	(17.09)	0.0163
Ferritin (ng/mL)	254.39	(1579.78)	174.46	(1310.87)	0.0551	254.39	(1579.78)	179.07	(768.93)	0.0606
Blood lead (μg/dL)	2.47	(3.30)	2.51	(2.79)	0.0149	2.47	(3.30)	2.65	(3.57)	0.0523
Blood zinc (μg/dL)	22.89	(37.31)	12.90	(29.25)	0.2979	22.89	(37.31)	18.53	(30.72)	0.1275

*Note*: This table summarises the baseline demographic, clinical and biochemical covariates comparing paediatric patients exposed to sevoflurane versus propofol. Standardised mean differences (ASMD) are provided before and after propensity score matching. Variables with ASMD ≥ 0.1 before matching indicate potential imbalance.

Abbreviations: 25‐OH vitamin D, 25‐hydroxyvitamin D; ASMD, absolute standardised mean difference; BMI, body mass index; free T4, free thyroxine; PSM, propensity‐score matching; SD, standard deviation; TSH, thyroid‐stimulating hormone.

**TABLE 2:** Incidence of ADHD and all‐cause mortality in children exposed to sevoflurane versus propofol after surgery (propensity‐score–matched cohorts)

Outcome	Cohort	Patients (*n*)	Events (*n*)	Risk (%)	Incidence rate (per 10 000 person‐years)	HR (95% CI)	RR (95% CI)	OR (95% CI)	Risk difference (95% CI)	*p* value
ADHD	Sevoflurane	27 051	1522	5.63	134.93	1.21 (1.11–1.31)	1.90 (1.75–2.07)	1.96 (1.79–2.14)	2.67% (2.33%–3.01%)	< 0.001
	Propofol	27 051	799	2.95	105.41	Ref.	Ref.	Ref.	Ref.	
Mortality	Sevoflurane	27 051	99	0.37	8.78	1.39 (0.98–1.97)	2.15 (1.52–3.05)	2.16 (1.52–3.06)	0.20% (0.11%–0.28%)	< 0.001
	Propofol	27 051	46	0.17	6.07	Ref.	Ref.	Ref.	Ref.	

*Note*: Analysis was performed in propensity‐score–matched paediatric cohorts (*n* = 27 051 each). Risk (%) represents Kaplan–Meier cumulative incidence estimates at the end of follow‐up. Incidence rates are expressed per 10 000 person‐years. Hazard ratios were estimated using Cox proportional hazards models with robust variance. Risk ratios and odds ratios were derived from cumulative incidence. Risk difference is expressed as absolute percentage points. Results for ADHD indicate that sevoflurane was associated with a higher risk relative to propofol, supporting the hypothesis that exposure to sevoflurane during paediatric surgery may increase the subsequent risk of ADHD. Mortality events were rare; the hazard ratio did not reach conventional significance, although the absolute risk difference was statistically significant.

Abbreviations: ADHD, attention‐deficit/hyperactivity disorder; CI, confidence interval; HR, hazard ratio; OR, odds ratio; Ref., reference group (propofol); RR, risk ratio.

## RESULTS

### Baseline characteristics

Before PSM, children exposed to sevoflurane were slightly older than those exposed to propofol (mean age, 4.9 vs. 4.3 years). The sevoflurane group included a higher proportion of Black or African American patients (22.85% [*n* = 6180] vs. 14.64% [*n* = 142 968]) and more children with chronic lower respiratory disease (9.88% [*n* = 2672] vs. 6.99% [*n* = 68 234]), perinatal conditions (8.13% [*n* = 2198] vs. 5.31% [*n* = 51 873]) and congenital malformations (25.64% [*n* = 6935] vs. 20.49% [*n* = 200 105]). In contrast, the propofol group had a greater proportion of Asian children (4.05% [*n* = 39 540] vs. 1.76% [*n* = 476]) and higher mean BMI (19.51 vs. 18.33 kg/m^2^). Blood zinc levels were also lower in the propofol group (12.90 vs. 22.89 μg/dL). After PSM, demographic, clinical and biochemical characteristics were well balanced between the two groups, with all standardised mean differences < 0.02, indicating successful covariate balance.

### Incidence of ADHD and mortality after sevoflurane versus propofol

In the PSM cohorts (27 051 children per group), the cumulative incidence of ADHD was significantly higher among children exposed to sevoflurane than among those who received propofol (5.63% vs. 2.95%), corresponding to incidence rates of 134.93 and 105.41 per 10 000 person‐years, respectively (table 2). Sevoflurane exposure was associated with a 21% increased hazard of ADHD (HR: 1.21; 95% CI 1.11–1.31; *p* < 0.001). Consistent results were observed in cumulative incidence analyses, where divergence between groups emerged early and widened over time (figure 3). The risk and odds ratios indicated nearly a doubling of risk, with an absolute risk difference of 2.67% points (95% CI 2.33–3.01).

Mortality was infrequent in both groups (0.37% vs. 0.17%), and the HR for death did not reach statistical significance (HR: 1.39; 95% CI 0.98–1.97). However, secondary measures indicated a modest absolute excess risk of death with sevoflurane (risk difference, 0.20%; 95% CI 0.11%–0.28%) (table 2). Survival curves confirmed the absence of a meaningful difference in overall survival despite the elevated ADHD risk (figure S2).

From an epidemiologic perspective, the alignment between point estimates, cumulative incidence curves and absolute risk differences provides convergent evidence that the observed association is not merely a statistical artefact but reflects a robust and clinically meaningful signal. The separation of ADHD incidence curves, coupled with the absence of confounding mortality differences, strengthens the inference that sevoflurane exposure itself may play a causal role in increasing ADHD risk among children.

### Subgroup analyses

In prespecified subgroup analyses (figure 1), the association between sevoflurane exposure and the subsequent risk of ADHD was broadly consistent across demographic and clinical strata. The excess risk was most evident among children 13–15 years of age (adjusted hazard ratio [aHR]: 1.38; 95% CI 1.15–1.64) and 16–18 years of age (aHR: 1.30; 95% CI 1.03–1.63), whereas estimates in younger age groups were closer to unity. Both boys and girls exhibited elevated risks, with no evidence of effect modification by sex. When stratified by race and ethnicity, the association was strongest among Black children (aHR: 1.47; 95% CI 1.23–1.75), although elevated risks were also observed in White and other racial groups. Analyses by the surgical category indicated that the increased risk was not confined to a single procedure type, with consistent estimates across musculoskeletal, digestive tract and urologic operations. Similarly, among children with preexisting respiratory, developmental or neurologic disorders, HRs remained elevated and closely aligned with those observed in the overall population. Taken together, the subgroup analyses strengthen the plausibility of our central hypothesis. The consistency of associations across age, sex, race, surgical categories, and baseline comorbidities suggests that the observed excess risk is unlikely to be explained by residual confounding within a particular high‐risk subgroup. The particularly elevated HRs among school‐aged children and Black children highlight populations in whom vulnerability to anaesthetic neurotoxicity may be greatest. Overall, the convergence of evidence across diverse strata supports the robustness of the primary analysis and indicates that sevoflurane exposure during paediatric anaesthesia may contribute to an increased risk of subsequent ADHD.

### Outcome validation and control analyses

In analyses of primary and secondary outcomes (figure 2), sevoflurane exposure was associated with a significantly increased risk of new‐onset ADHD as compared with propofol (aHR: 1.21; 95% CI 1.11–1.31). The HR for all‐cause mortality was higher in the sevoflurane group, although the CI crossed unity (aHR: 1.39; 95% CI 0.98–1.97). Among specificity outcomes, the risk of anxiety disorders was elevated (aHR: 1.33; 95% CI 1.25–1.42), whereas no association was observed for depressive disorders (aHR: 0.96; 95% CI 0.85–1.09). For positive control outcomes, the association with delirium was directionally consistent but imprecise (aHR: 1.16; 95% CI 0.66–2.03). Negative control outcomes, including common cold and irritable bowel syndrome, showed no clear associations, with estimates clustered around the null (aHR: 1.15; 95% CI 1.00–1.30; and aHR: 1.00; 95% CI 0.77–1.29, respectively).

### Sensitivity analyses

In multiple sensitivity analyses, the association between sevoflurane exposure and subsequent ADHD proved to be consistent and robust. When follow‐up was redefined to begin 18 or 24 months after anaesthesia to minimise perioperative ascertainment bias, the HRs were modestly attenuated but remained significant (HRs: 1.18 and 1.15, respectively) (table S1). Restriction to children with at least 2 or 3 years of continuous observation yielded nearly identical results (HRs: 1.20 and 1.19) (table S2). Alternative propensity‐score strategies, including narrower callipers and trimming of extreme scores, also produced estimates in the same range (HRs: 1.18–1.21), with good covariate balance achieved in all models (ASMD < 0.1) (table S3).

Further strengthening diagnostic specificity, HRs were slightly higher when ADHD was defined by multiple coded encounters, stimulant prescriptions or psychiatric confirmation (HRs: 1.23–1.26) (table S4). Exclusion of children with congenital anomalies, epilepsy or severe perinatal complications did not materially change the estimates (HRs: 1.19–1.20) (table S5). Finally, quantitative bias analyses indicated that an unmeasured confounder would need to be associated with both exposure and outcome with a risk ratio of at least 1.7 (and 1.45 for the lower bound of the CI) to fully account for the observed association (table S6).

## DISCUSSION

### Main findings

In this large, multinational cohort of more than 54 000 PSM children, real‐world observational data showed that exposure to sevoflurane during surgery was associated with a significantly higher subsequent risk of ADHD than exposure to propofol, without implying causality. The cumulative incidence of ADHD was 5.63% in the sevoflurane group and 2.95% in the propofol group (table 2), corresponding to an absolute risk difference of 2.67% (95% CI 2.33%–3.01%). This translated into an HR of 1.21 (95% CI 1.11–1.31), with relative risk and odds ratios indicating nearly a doubling of risk. Kaplan–Meier analyses (figure 3) showed early separation of incidence curves, with progressive divergence over time, suggesting a sustained impact of anaesthetic choice on long‐term neurodevelopmental outcomes. Baseline characteristics were well balanced after matching, with all standardised mean differences below 0.02 (table 1), indicating that the observed association is unlikely to be explained by residual confounding in measured covariates. Mortality was rare in both groups, and although the HR for death was slightly elevated with sevoflurane (HR: 1.39; 95% CI 0.98–1.97), survival curves demonstrated no meaningful difference in overall survival (figure S2). Collectively, these findings establish a robust and clinically relevant signal: sevoflurane exposure was consistently associated with excess risk of ADHD, whereas propofol appeared comparatively safer in this paediatric surgical population. This investigation represents the first large‐scale, multinational analysis to directly compare sevoflurane and propofol with respect to long‐term neurodevelopmental outcomes in children. By leveraging a global federated electronic health record network and applying rigorous PSM, we provide evidence that addresses a long‐standing uncertainty in clinical practice. Importantly, these results shift the debate from whether anaesthesia in general contributes to ADHD risk to identifying which specific agents may confer that risk, thereby offering actionable insights to inform anaesthetic selection and guide perioperative counselling for families.

### Comparison with prior clinical evidence

Our findings extend a substantial body of preclinical and clinical evidence suggesting that volatile anaesthetics, particularly sevoflurane, may exert long‐term neurodevelopmental effects. Experimental studies across animal models have consistently demonstrated that exposure to volatile agents during critical periods of brain maturation induces widespread neuronal apoptosis, neuroinflammation and lasting cognitive and behavioural impairments.^6^, ^11^, ^30^ Sevoflurane, in particular, has been shown to alter NMDA receptor subunit composition and disrupt synaptic plasticity within the developing prefrontal cortex (PFC), whereas propofol—with its rapid metabolism and predominant GABA_A_ receptor modulation—appears to produce less sustained neurotoxicity under comparable experimental conditions.^31^, ^32^ These mechanistic distinctions provide biological plausibility for the differential outcomes observed in our clinical data.^6^, ^11^, ^30^, ^31^, ^32^ Clinical evidence prior to this study has been limited and often inconclusive.^13^, ^14^, ^15^, ^16^, ^17^, ^18^, ^19^ Most studies were small, lacked anaesthetic granularity and often grouped all general anaesthetics together without distinguishing between agents.^33^, ^34^, ^35^, ^36^ Large‐scale randomised or matched cohort studies, including the General Anaesthesia Compared to Spinal anaesthesia Trial, Pediatric Anesthesia NeuroDevelopment Assessment Study and Mayo Anesthesia Safety in Kids Study, provided reassuring evidence that single, brief exposures to general anaesthesia do not impair global intelligence.^37^, ^38^, ^39^ However, these investigations were not designed to examine agent‐specific risks, and most excluded children undergoing repeated or complex procedures—precisely those at higher neurodevelopmental vulnerability.^37^, ^38^, ^39^ Recent work in *General Psychiatry* has further refined the conceptualisation of ADHD as a disorder of altered neurodevelopmental network integration and attentional control, characterised by disrupted information processing and cortical network dynamics rather than isolated cognitive deficits.^12^ Consequently, whether specific anaesthetics differentially influence behavioural and attentional outcomes has remained an unresolved question in paediatric anaesthesia. The present multinational cohort study directly addresses this knowledge gap by performing a large‐scale, head‐to‐head comparison between sevoflurane and propofol, using a rigorously matched real‐world dataset encompassing more than 54 000 children across 150 institutions worldwide. Through robust PSM, multiple sensitivity analyses and validation against both positive and negative control outcomes, this study provides the strongest epidemiologic evidence to date that sevoflurane exposure is associated with an increased long‐term risk of ADHD, whereas propofol is not. In doing so, it shifts the field from asking whether anaesthesia per se contributes to neurodevelopmental risk towards identifying which agents may drive that association—offering a more actionable foundation for clinical decision‐making and family counselling.

### Biological plausibility and mechanistic interpretation

The observed association between sevoflurane exposure and increased ADHD risk is biologically plausible, reflecting multiple converging pathways of neurotoxicity in the developing brain. Volatile anaesthetics such as sevoflurane act as dual modulators—inhibiting NMDA receptors while enhancing GABA_A_ receptor activity.^7^, ^8^, ^11^, ^23^ This dual mechanism has been shown in animal models during the synaptogenesis period to trigger widespread neuronal apoptosis, disruption of synaptic plasticity and aberrant remodelling of prefrontal cortical circuits, all of which parallel the neurobiological features of ADHD.^7^, ^8^, ^11^, ^23^ Experimental studies have demonstrated that repeated early life exposure to sevoflurane delays the developmental switch of NMDA receptor subunits (from NR2B to NR2A) and increases NR1/NR2B expression in the PFC, resulting in persistent deficits in attention and working memory regulation.^31^, ^40^, ^41^, ^42^ Moreover, sevoflurane has been shown to induce oxidative stress, neuroinflammation and dendritic spine loss, providing additional mechanistic links between anaesthetic exposure and long‐term neurobehavioral outcomes.^43^, ^44^ By contrast, propofol primarily acts as a selective GABA_A_ receptor agonist with minimal NMDA receptor interference. Its short duration of action and rapid metabolism limit neuronal exposure time, and its documented antioxidant and anti‐inflammatory properties may further mitigate potential neurotoxic effects.^24^, ^25^, ^45^ In parallel, population‐based psychiatric research has demonstrated substantial comorbidity and shared vulnerability between ADHD and other neurodevelopmental or neurologic conditions with genetic correlation insufficient to fully explain observed associations, highlighting the potential role of early environmental or iatrogenic exposures.^46^ Taken together, the mechanistic evidence supports a biologically credible hypothesis: Sevoflurane may perturb neurodevelopment through NMDA/GABA imbalance, oxidative stress and neuroinflammatory cascades, leading to alterations in cortical maturation and increased vulnerability to ADHD in later childhood.

### Robustness of findings and sensitivity analyses

The internal validity of our findings was strengthened through multiple complementary sensitivity analyses designed to address potential sources of bias. Across a broad range of specifications—including lag‐time analyses redefining follow‐up initiation at 18 and 24 months after exposure, minimum observation‐period restrictions requiring at least 2 or 3 years of continuous follow‐up and alternative ADHD case definitions (e.g., two or more diagnostic codes, stimulant prescriptions, or psychiatric confirmation)—the aHRs remained remarkably stable (tables S1–S4). Exclusion of children with congenital, epileptic or perinatal disorders did not materially change the estimates, suggesting that the association was not driven by preexisting neurodevelopmental vulnerability (table S5). Moreover, a quantitative bias analysis indicated that an unmeasured confounder would need to be associated with both exposure and outcome by a risk ratio ≥ 1.7 to fully explain the observed association, supporting the causal robustness of the signal (table S6). We further implemented positive and negative outcome controls to evaluate residual and detection biases. Delirium, prespecified as a positive control outcome, showed a directionally consistent but imprecise association, whereas the null findings for common cold and irritable bowel syndrome provided reassurance that the observed ADHD association was not due to nonspecific diagnostic or surveillance bias. Collectively, these multi‐level sensitivity checks, alongside rigorous PSM with near‐perfect covariate balance (standardised mean differences < 0.02), demonstrate the methodologic rigour and internal consistency of our findings. Taken together, the convergence of evidence across analytic frameworks substantiates the robustness of our conclusion that sevoflurane exposure in childhood is consistently associated with an elevated long‐term risk of ADHD.

### Limitations

This study should be interpreted in the context of several limitations inherent to observational pharmacoepidemiologic research. First, although residual confounding cannot be completely excluded in a nonrandomised design, we implemented rigorous PSM, positive and negative control outcomes and multiple sensitivity analyses to mitigate both measured and unmeasured biases. The use of a large, multinational real‐world database ensured sufficient statistical power and generalisability across diverse populations, whereas standardised diagnostic and procedural codes enhanced comparability between exposure groups. Although potential misclassification of ADHD diagnoses and anaesthetic exposures remains possible, diagnostic specificity was strengthened by requiring repeated ADHD codes and stimulant prescriptions in sensitivity analyses. Furthermore, although granular details such as anaesthetic dose and duration were unavailable, restriction to single‐exposure cases minimised bias related to cumulative or repeated anaesthesia. In addition, detailed and consistently reliable data on postoperative analgesic exposure, including opioid use (e.g., fentanyl), were not available across institutions and therefore could not be quantitatively adjusted, representing a potential source of nondifferential residual confounding. Future prospective studies incorporating standardised anaesthetic dosing records and intraoperative depth‐of‐anaesthesia monitoring (e.g., minimum alveolar concentration or bispectral index) would be valuable to further explore potential dose–response or threshold effects. Finally, quantitative bias analysis indicated that an unmeasured confounder would need to be strongly associated with both exposure and outcome (risk ratio ≥ 1.7) to fully account for the observed association, underscoring the robustness of our findings.

### Implications

From a clinical standpoint, these results have important implications for paediatric anaesthetic practice. The consistent association between sevoflurane exposure and elevated ADHD risk—especially among school‐aged children and those with developmental vulnerabilities—suggests that anaesthetic choice during early neurodevelopmental periods may warrant heightened caution. Although propofol remains an alternative with a more favourable neurobiologic profile, anaesthetic selection should continue to balance procedural feasibility, haemodynamic stability and long‐term neurocognitive safety. Future research should aim to confirm these findings through prospective cohort studies and mechanistic investigations integrating neuroimaging, electrophysiologic and molecular biomarkers to clarify the causal pathways linking anaesthetic exposure to neurodevelopmental outcomes. Such work will be essential to refine perioperative counselling, inform anaesthesia stewardship policies and ultimately optimise neurodevelopmental safety in children undergoing surgery.

## CONCLUSIONS

This multinational study provides the first real‐world, head‐to‐head evidence that the choice of anaesthetic agent—not anaesthesia exposure per se—may determine long‐term neurodevelopmental risk in children. By demonstrating a consistent association between sevoflurane exposure and elevated ADHD incidence, our findings challenge the prevailing assumption that all anaesthetic agents are neurodevelopmentally equivalent and highlight the need for agent‐specific neurotoxicity consideration in paediatric anaesthesia. These results bridge a long‐standing gap between experimental and clinical data, offering an actionable framework for anaesthetic selection, perioperative counselling and policy development to enhance neurodevelopmental safety in children worldwide.

## AUTHOR CONTRIBUTIONS

Mingyang Sun and Yangyang Wang contributed equally to this work. They were responsible for the study conception, design, data collection and drafting of the initial manuscript. Zhongyuan Lu and Jiao Wang contributed to data curation, statistical analysis and interpretation of findings. Yuqin Tang and Mengrong Miao assisted with patient recruitment, clinical data acquisition and the validation of results. Wan‐Ming Chen provided methodological guidance, critical revision of the manuscript and supervision of analytic approaches. Szu‐Yuan Wu served as senior author, supervised the study design and execution, provided major intellectual input and critically revised the manuscript for important intellectual content. Jiaqiang Zhang served as senior author, contributed to overall study conception and design, provided oversight of clinical components and critically revised the manuscript. Jiaqiang Zhang serves as the guarantor of this study, and, as such, has full access to all the data in the study. He takes responsibility for the integrity of the data and confirms that all research data are authentic, accurate and free from fabrication or falsification and that the manuscript represents an honest, accurate and transparent account of the study.

## CONFLICT OF INTEREST STATEMENT

The authors declare no conflicts of interest.

## ETHICS APPROVAL AND CONSENT

This study utilised the TriNetX platform, which exclusively provides de‐identified and aggregated electronic health record data. In accordance with the Health Insurance Portability and Accountability Act (HIPAA), such data do not allow for individual identification and thus do not meet the federal definition of human subject research. Consequently, research conducted using this platform is exempt from Institutional Review Board (IRB) oversight and does not require informed consent.

## Supporting information

Supporting Information S1

Supporting Information S2

## Data Availability

The data that support the findings of this study are available from the TriNetX Global Collaborative Network. These data are provided in a de‐identified and aggregated format and are not publicly available due to data use agreements and privacy restrictions. Researchers with appropriate approvals may access the data through the TriNetX platform (https://trinetx.com), subject to institutional agreements and compliance with applicable data protection regulations.
