# Supplementary material for: Sevoflurane versus propofol and the long‐term risk of attention‐deficit/hyperactivity disorder in children
Source: Gen Psychiatr. 2026 Apr 16;39(2):e70000. doi: 10.1002/gps3.70000 (PMC13084326; doi:10.1002/gps3.70000)

**Supplemental Figure 1. Study flow-chart**

Children and adolescents aged 0–18 years who underwent surgical procedures requiring general anesthesia identified in the TriNetX Global Collaborative Network between September 2005 and September 2025. (N = 1 425 139)

(N = 1,424,527)

Exclusion Criteria (Total N = 421 612)

1. Patients with < 12 months of continuous medical records before the index date (N = 154 892).
2. Patients with a prior diagnosis of ADHD (N = 65 214).
3. Patients who received both Sevoflurane and Propofol on the index date (N = 28 043).
4. Patients who underwent more than one qualifying surgery during the study period (N = 120 657).
5. Patients with incomplete demographic or baseline information (N = 34 921).
6. Patients with less than 6 months of follow-up after the index date (N = 17 885).

Patients meeting inclusion criteria for single anesthetic exposure (N = 1 003 527).

Propensity Score Matching performed at a 1:1 ratio to adjust for confounders: Age, sex, race, ethnicity, comorbidities (chronic respiratory diseases, epilepsy, sleep disorders, congenital malformations, perinatal conditions, psychiatric disorders), and biomarkers (BMI, thyroid function, vitamin D, ferritin, blood lead, zinc, magnesium).

Sevoflurane Group

(N = 27 051)

Propofol Group

(N = 27 051)

Comparison Group

Case Group

**Supplemental Figure 2. Overall Survival in Children Receiving Sevoflurane versus Propofol Showing No Mortality Difference Despite Increased Risk of Attention-Deficit/Hyperactivity Disorder with Sevoflurane.**


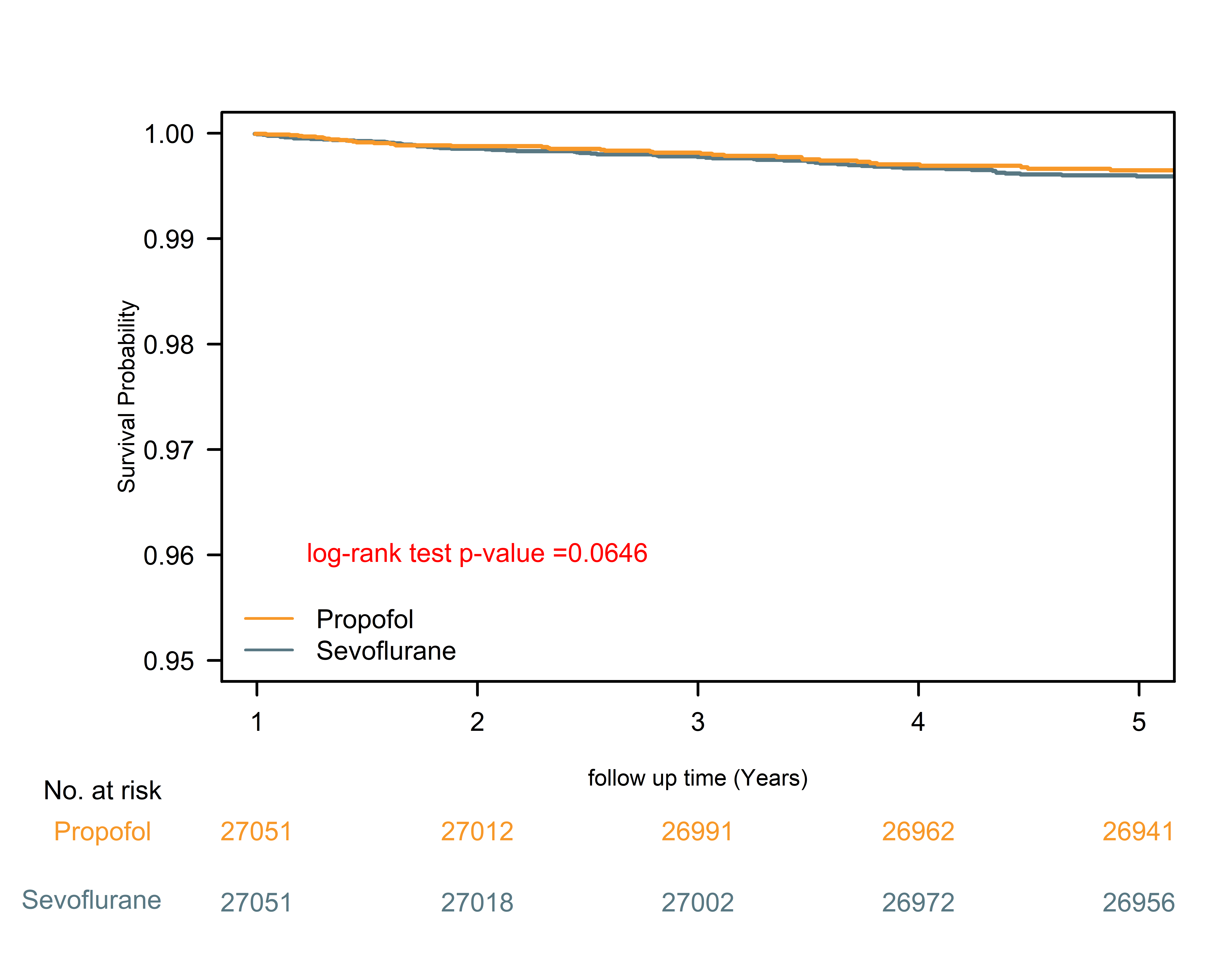

Supplement: Supplementary file 1 — Supporting Information S1 [file GPS3-39-e70000-s001.docx]
