# Supplementary material for: Sevoflurane versus propofol and the long‐term risk of attention‐deficit/hyperactivity disorder in children
Source: Gen Psychiatr. 2026 Apr 16;39(2):e70000. doi: 10.1002/gps3.70000 (PMC13084326; doi:10.1002/gps3.70000)
Supplement: Supplementary file 2 — Supporting Information S2 [file GPS3-39-e70000-s002.docx]

## **Online Supplementary Table** 1. Lag-Time Sensitivity Analyses for ADHD (Follow-up Initiation at +18 and +24 Months)

| **Follow-up Start** | **Cohort** | **Patients (N)** | **Events (N)** | **Risk (%)** | **HR (95% CI)** | **p-value** |
| --- | --- | --- | --- | --- | --- | --- |
| **+18 months** | Sevoflurane | 27 051 | 1 372 | 5.07 | — | — |
| **+18 months** | Propofol | 27 051 | 713 | 2.64 | 1.18 (1.08–1.29) | 0.0004 |
| **+24 months** | Sevoflurane | 27 051 | 1 245 | 4.60 | — | — |
| **+24 months** | Propofol | 27 051 | 648 | 2.39 | 1.15 (1.04–1.27) | 0.0058 |

Abbreviations: HR = hazard ratio; CI = confidence interval.
Footnote: Follow-up start was delayed to +18 or +24 months post-index to mitigate perioperative ascertainment bias.

## **Online Supplementary Table** 2. Sensitivity Analyses by Minimum Continuous Follow-up (≥2 Year and ≥3 Years)

| **Restriction** | **Cohort** | **Patients (N)** | **Events (N)** | **Risk (%)** | **HR (95% CI)** | **p-value** |
| --- | --- | --- | --- | --- | --- | --- |
| **≥2 years** | Sevoflurane | 21 934 | 1 186 | 5.41 | — | — |
| **≥2 years** | Propofol | 21 947 | 619 | 2.82 | 1.20 (1.09–1.31) | <0.001 |
| **≥3 years** | Sevoflurane | 16 823 | 891 | 5.30 | — | — |
| **≥3 years** | Propofol | 16 845 | 456 | 2.71 | 1.19 (1.06–1.33) | 0.003 |

Abbreviations: HR = hazard ratio; CI = confidence interval.
Footnote: Analyses restricted to patients with continuous observation ≥365 or ≥730 days.

## **Online Supplementary Table** 3. Alternative Propensity Score–Matching Specifications

| **PS Strategy** | **ASMD Post-adjustment (Median/IQR)** | **Effective Sample Size** | **ADHD HR (95% CI)** | **p-value** |
| --- | --- | --- | --- | --- |
| **Caliper 0.20 (current)** | <0.10 (balanced) | 27 051 vs 27 051 | 1.21 (1.11–1.31) | <0.001 |
| **Caliper 0.10** | <0.08 (0.04–0.10) | 25 832 vs 25 817 | 1.19 (1.09–1.31) | <0.001 |
| **PS overlap trimming (1–5%)** | <0.07 (0.04–0.09) | 23 964 vs 23 951 | 1.18 (1.07–1.30) | 0.001 |

Abbreviations: PSM = propensity score matching; ASMD = absolute standardized mean difference; HR = hazard ratio; CI = confidence interval.
Footnote: Re-matching with stricter calipers and PS overlap trimming to assess robustness; balance by ASMD.

## **Online Supplementary Table** 4. Sensitivity Analyses Under Alternative ADHD Case Definitions

| **ADHD Definition** | **Cohort** | **Patients (N)** | **Events (N)** | **Risk (%)** | **HR (95% CI)** | **p-value** |
| --- | --- | --- | --- | --- | --- | --- |
| **≥2 ADHD-coded encounters** | Sevoflurane | 27 051 | 1 058 | 3.91 | — | — |
| **≥2 ADHD-coded encounters** | Propofol | 27 051 | 541 | 2.00 | 1.23 (1.10–1.38) | <0.001 |
| **ADHD + stimulant prescription** | Sevoflurane | 27 051 | 781 | 2.89 | — | — |
| **ADHD + stimulant prescription** | Propofol | 27 051 | 392 | 1.45 | 1.25 (1.09–1.42) | 0.001 |
| **ADHD in child psychiatry encounter** | Sevoflurane | 27 051 | 601 | 2.22 | — | — |
| **ADHD in child psychiatry encounter** | Propofol | 27 051 | 302 | 1.12 | 1.26 (1.07–1.49) | 0.006 |

Abbreviations: ADHD = attention-deficit/hyperactivity disorder; HR = hazard ratio; CI = confidence interval.

## **Online Supplementary Table** 5. Sensitivity Analyses Excluding Children with Baseline Neurodevelopmental Vulnerabilities

| **Exclusion Set** | **Cohort** | **Patients (N)** | **Events (N)** | **Risk (%)** | **HR (95% CI)** | **p-value** |
| --- | --- | --- | --- | --- | --- | --- |
| **Exclude congenital/chromosomal** | Sevoflurane | 19 984 | 1 019 | 5.10 | — | — |
| **Exclude congenital/chromosomal** | Propofol | 19 976 | 541 | 2.71 | 1.20 (1.08–1.34) | 0.001 |
| **Exclude epilepsy & seizures** | Sevoflurane | 25 934 | 1 446 | 5.57 | — | — |
| **Exclude epilepsy & seizures** | Propofol | 25 927 | 758 | 2.92 | 1.19 (1.09–1.31) | <0.001 |
| **Exclude perinatal complications** | Sevoflurane | 25 472 | 1 358 | 5.33 | — | — |
| **Exclude perinatal complications** | Propofol | 25 460 | 719 | 2.82 | 1.20 (1.09–1.33) | <0.001 |

Abbreviations: HR = hazard ratio; CI = confidence interval.
Footnote: Exclusions applied prior to matching; cohorts re-matched and Cox models refit.

## **Online Supplementary Table** 6. Quantitative Bias Analyses: E-values

| **Outcome / Estimate** | **Value** |
| --- | --- |
| ADHD – HR (point estimate = 1.206) | E-value = 1.70 |
| ADHD – HR (95% CI lower bound = 1.107) | E-value = 1.45 |
| Mortality – HR (point estimate = 1.390) | — |
| Mortality – HR (95% CI lower bound = 0.979) | — |

Abbreviations: HR = hazard ratio; CI = confidence interval.
Footnote: E-values quantify the minimum association strength an unmeasured confounder must have with both exposure and outcome (on the risk ratio scale) beyond measured covariates to fully explain away the observed association.
